# A survey of East Palaearctic Gnaphosidae (Araneae). 2. Two new *Gnaphosa* Latreille, 1804 species from Western Mongolia

**DOI:** 10.3897/zookeys.426.7898

**Published:** 2014-07-17

**Authors:** Yuri M. Marusik, Alexander A. Fomichev, Mikhail M. Omelko

**Affiliations:** 1 Institute for Biological Problems of the North, RAS, Portovaya Str. 18, Magadan RF-685000, Russia; 2 Zoological Museum, University of Turku, FI-20014 Turku, Finland; 3 Altai State University, Lenina Pr., 61, Barnaul, RF-656049, Russia; 4 Gornotaezhnaya Station FEB RAS, Gornotaezhnoe Vil., Ussuriysk Dist., Primorski Krai RF-692533, Russia; 5 Far Eastern Federal University, Sukhanova 8, Vladivostok RF-690950, Russia

**Keywords:** Spiders, Gnaphosinae, Mongolia, new species, taxonomy

## Abstract

Two new species: *Gnaphosa khovdensis* **sp. n.** (♂) and *G. esyunini* **sp. n.** (♂♀) are described from Khovd Aimag of Mongolia. Descriptions, figures, diagnosis and map of the records are provided. The new species are assigned to a monophyletic group together with the recently described *G. ustyuzhanini* Fomichev et al., 2013.

## Introduction

*Gnaphosa* Latreille, 1804 is the third largest genus of ground spiders with 141 species (Platnick 2014) and is one of the best-studied species rich genera of Holarctic spiders. The genus occurs from south China (Song et al. 2004) to the high Arctic reaching 71°34'N in Greenland (Marusik et al. 2006) and 72°15'N in Asia (Marusik et al. 1993). The genus has been well studied in wide scale revisions of the Nearctic (Platnick and Shadab 1975), European (Grimm 1985), Asian (Ovtsharenko et al. 1992) and Chinese (Song et al. 2004) species.

While studying material collected in Western Mongolia and particularly in Khovd Aimag we recognized two unknown species related to each other and to the recently described *Gnaphosa ustyuzhanini* Fomichev et al., 2013. Goals of this paper are to provide detailed descriptions of the new species and to discuss their relationships.

## Material and methods

Photographs were taken in dishes of different sizes with paraffin at the bottom. Specimens were photographed using an Olympus Camedia E-520 camera attached to an Olympus SZX16 stereomicroscope and the SEM JEOL JSM-5200 scanning microscope at the Zoological Museum, University of Turku. Digital images were prepared using “CombineZP” image stacking software. Illustrations of epigynes were made after maceration in 20% potassium hydroxide aqueous solution and exposition for a few minutes in an alcohol/water solution of Chlorazol Black. Lengths of leg segments were measured on the dorsal side. While describing spination of legs we use the following abbreviations: Fe – femur, Pt – patella, Ti – tibia, Mt – metatarsus. All measurements are given in mm. Material will be deposited in the Institute for Systematic and Ecology of Animals, Novosibirsk (ISEA), Institute of Zoology, Chinese Academy of Sciences, Beijing (IZCAS) and in the Museum of Natural History, Budapest (MNHB).

## Species survey

### Gnaphosa khovdensis sp. n.

Taxon classificationAnimaliaAraneaeGnaphosidae

http://zoobank.org/03D9E1C9-6024-41AA-9049-4CB538E6C52C

Figs 1–6

#### Material.

MONGOLIA, ***Khovd*** Aimag: holotype ♂ (ISEA), Arshantyn-Nuruu Mountain Range, 46°16'46"N, 91°16'53"E, 1560 m, mountain stony steppe, under stone, 14.05.2012 (A.A. Fomichev).

#### Etymology.

The specific name derived from Khovd Aimag, adjective.

#### Diagnosis.

The new species is related to *Gnaphosa esyunini* sp. n., *Gnaphosa jucunda* Thorell, 1875 and *Gnaphosa ustyuzhanini* by having a strong spine in the embolic base and a filamentous embolus with a serrated prolateral edge. From *Gnaphosa ustyuzhanini* (Figs 17–18) and *Gnaphosa jucunda* it can be distinguished by the prolaterally directed strong embolic spine. In addition *Gnaphosa jucunda* has a thick, not filamentous embolus (cf. Ponomarev and Kovblyuk 2009: figs 19–20) and smaller median apophysis. *Gnaphosa khovdensis* sp. n. differs from *Gnaphosa esyunini* sp. n. by the smaller embolic spine located on the retrolateral side of the embolus and smaller body size.

#### Description.

Male. Total length 8.5. Carapace: 4.05 long, 3.05 wide. Coloration: carapace and legs brown. Chelicerae dark brown. Sternum, labium and maxillae brown. Abdomen grayish-brown. Spinnerets light brown. Spination: I: Fe d1-1-0, p0-1-1; Ti v0-1-1; Mt v2-1-0. II: Fe d1-1-0, p0-1-1; Ti p1-0-0, v1-1-2; Mt v2-1-0. III: Fe d1-1-0, p0-1-1, r0-1-1; Pt p1, r1; Ti d1-0-0, p2-2-0, r2-1-1, v2-2-2; Mt d0-2-0, p1-1-0, r1-1-0, v2-2-0. IV: Fe d1-1-0, p0-1-1, r0-1-1; Pt r1; Ti p2-2-0, r2-1-1, v2-2-2; Mt d0-2-0, p1-1-0, r1-1-0, v2-2-0. Leg article length. I: 3.15+1.7+2.65+2.3+1.65. II: 3.0+1.6+2.4+2.3+1.65. III: 2.7+1.35+2.0+2.6+1.65. IV: 3.55+1.7+2.9+3.9+2.0.

**Figures 1–6.**
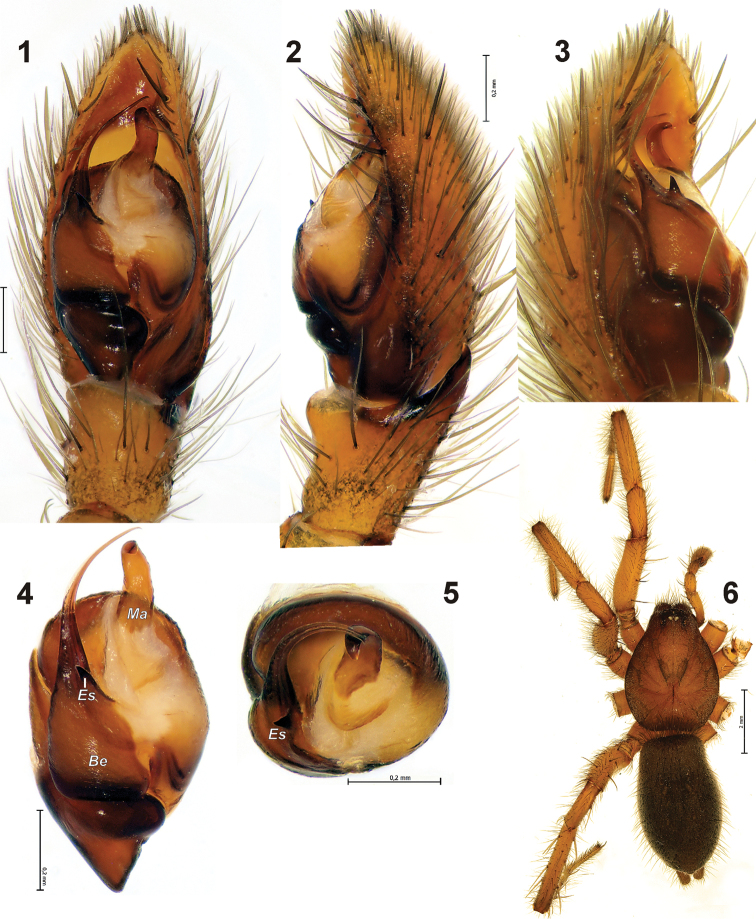
Holotype of *Gnaphosa khovdensis* sp. n. **1–3** male palp, ventral, retro and prolateral **4–5** bulbus, ventral and from above **6** habitus. Scale = 0.2 mm if not otherwise indicated. *Be* – base of embolus; *Es* – embolic spine; *Ma* – median apophysis.

Palp as in Figs 1–5, tibial apophysis relatively short (shorter than tibia) with sharply pointed tip bent downward; median apophysis relatively small, as long as tibia; embolus unmodified, whip like, base of embolus located on posterior 1/3 of bulbus; upper part of embolic base with beak-shaped spine; prolateral edge of embolus serrated.

Female unknown.

#### Distribution.

Known from the type locality only (Fig. 19).

### Gnaphosa esyunini sp. n.

Taxon classificationAnimaliaAraneaeGnaphosidae

http://zoobank.org/E03DBE63-88A9-4231-8F39-38DEA30D3CAF

Figs 7–16

#### Material.

MONGOLIA, ***Khovd*** Aimag: Holotype ♂ (NHMB) and paratypes 2♀ (NHMB) with label “Nr. 666. Chovd aimak: Chovd (Kobdo), ca 5 km SW von der Stadt, 1500 m, 10.VII.1966. – Von kahlen, felsigen Bergen umgegebener Tal mit sandigem Schotter, geeinzelt unter Steinen, vom Boden und zwischen den Pflansenwurzeln, sowie von den Pflanzen”; 1 ♀ (IZCAS), collecting number: mk408, 18.5 km of Monhhairhan Town, Monhhairhan Sum, Khovd Aimag, Mongolia, 47.22783°N, 91.89552°E, 1945 m, 22.07.2011 (Meng Kaibayier).

#### Note.

Different maps provide different spelling of Monhhairhan, one alternative spelling is Monkh Khayrkhan.

#### Etymology.

The species is named after our colleague Sergei L. Esyunin from Perm University (Russia), noun.

#### Diagnosis.

Males of the new species differ from all congeners by the large embolic spine directed prolaterally. Females of *Gnaphosa esyunini* sp. n. substantially differ from other species by the very wide fovea and the shape and angle of the lateral pockets.

#### Description.

Male. Total length 14.4. Carapace: 7.3 long, 5.6 wide. Coloration: carapace and legs brown. Labium, maxillae and sternum dark brown. Chelicerae dark brown, almost black. Abdomen grayish-brown. Spinnerets light brown. Spination: I: Fe d1-1-0, p0-0-1; Ti v0-1-1; Mt v0-2-0. II: Fe d1-1-0, p0-1-1; Ti v0-0-1; Mt v0-2-0. III: Fe d1-1-0, p0-1-1, r0-1-1; Pt p1, r1; Ti d1-0-0, p3-1-1, r2-1-1, v2-2-2; Mt d0-2-0, p2-1-0, r1-1-0, v0-3-2. IV: Fe d1-1-0, p0-1-1, r0-1-1; Pt r1; Ti p2-3-3, r3-2-3, v4-1-4; Mt d0-3-0, p3-4-1, r3-1-0, v3-3-3. Leg article length. I: 5.7+3.3+5.25+4.4+2.65. II: 5.3+3.0+4.75+4.4+2.75. III: 4.85+2.5+3.85+5.05+2.65. IV: 6.0+3.0+5.2+7.25+3.05.

**Figures 7–12.**
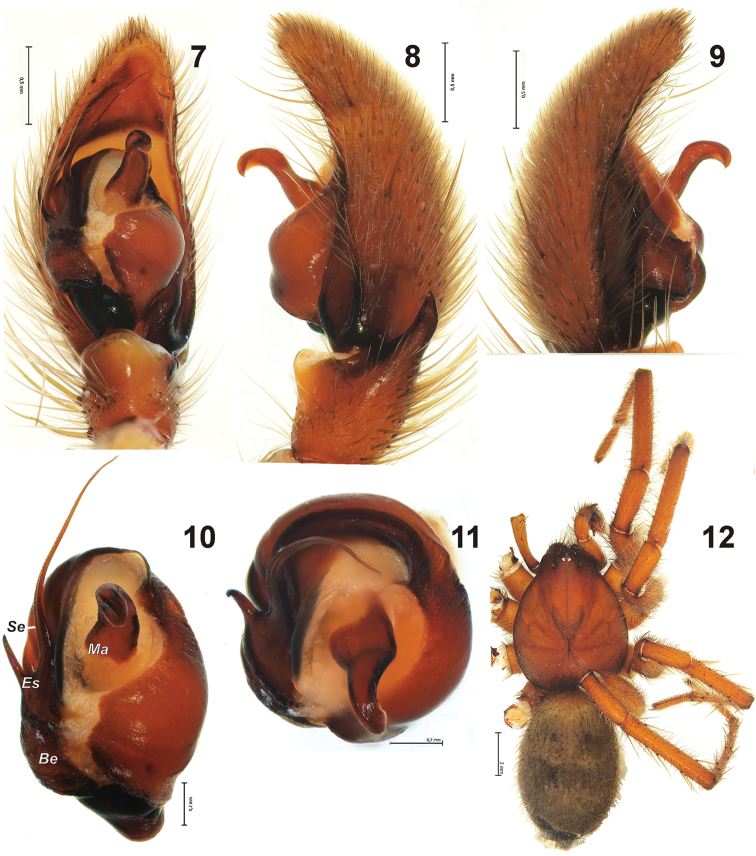
Holotype of *Gnaphosa esyunini* sp. n. **7–9** male palp, ventral, retro and prolateral **10–11** bulbus, ventral and from above **12** habitus. Scale = 0.2 mm if not otherwise indicated. *Be* – base of embolus; *Es* – embolic spine; *Ma* – median apophysis; *Se* – serrated edge of embolus.

Palp as in Figs 7–11; tibial apophysis with parallel margins and triangle-shaped tip; median apophysis relatively large and strong, partly turned due to inflation of distal haematodocha; base of embolus with long spine directed prolaterally and small teeth below the spine; prolateral edge of embolus distinctly serrated.

Female. Total length: 15.5. Carapace: 8.3 long, 6.15 wide. Coloration as in male. Spination: I: Fe d1-1-0, p0-0-1; Ti v0-1-1; Mt v0-2-0. II: Fe d1-1-0, p0-1-1; Ti p0-0-1, v0-1-2; Mt p1-0-0, v0-2-0. III: Fe d1-1-0, p0-1-1, r0-1-1; Pt p1, r1; Ti d2-0-0, p3-1-1, r2-1-1, v2-2-2, Mt d0-2-0, p2-1-0, r2-1-0, v2-0-2. IV: Fe d1-1-0, p0-1-1, r0-1-1; Pt r1; Ti p3-1-1, r2-1-2, v2-3-2; Mt d0-2-0, p2-2-0, r2-1-1, v2-1-2. Leg length. I: 5.9+3.6+4.9+3.95+2.8. II: 5.55+3.35+4.55+4.0+2.8. III: 5.05+2.75+3.8+4.7+2.8. IV: 6.15+3.3+5.3+7.1+3.2.

**Figures 13–16.**
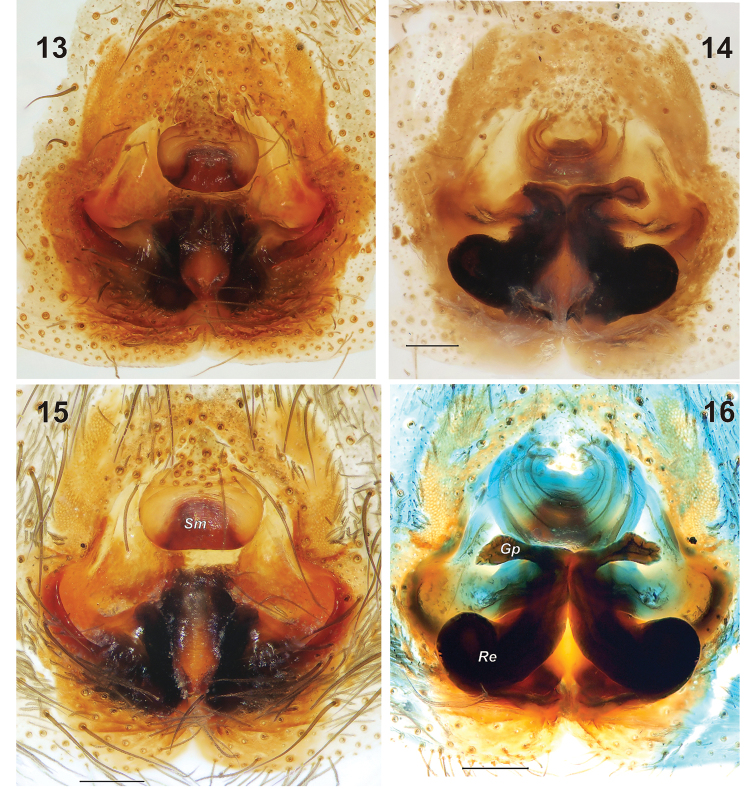
Epigyne of *Gnaphosa esyunini* sp. n. **13, 15** ventral **14, 16** dorsal, after maceration. Scale = 0.2 mm. *Gp* – glandular part of receptacle; *Re* receptacle; *Sm* – scape.

Epigyne as in Figs 13–16; fovea very wide, especially in posterior half, 2.2 times wider than scape; scape oval, wider than long, with large hood; lateral pockets long, at an angle of about 45° to epigastral fold; glandular part of receptacles stretched horizontally.

#### Distribution.

Known only from two localities in Khovd Aimag (Fig. 19).

### Gnaphosa ustyuzhanini

Taxon classificationAnimaliaAraneaeGnaphosidae

Fomichev, Marusik & Omelko, 2013

Figs 17–18

Gnaphosa ustyuzhanini Fomichev et al. 2013: 153, f. 1–10 (♂).

#### Material examined.

MONGOLIA: ***Khovd*** Aimag: 1♂ (NHMB) with label “Nr.649, Z.Kaszab, Chovd aimak: Mongol Altaj Gebirge, Tal des Flusses Uenč gol, cca 64 km N von Somon Uenč, 2100 m, 8.VII.1966. – Im Flusstal unter Steinen geeinzelt, resp. geschossen.”

#### Comments.

The type series of this species was collected at the same place where the type locality of *Gnaphosa khovdensis* sp. n. is located. The newly examined specimen has a more prominent embolic spine than in the holotype and paratype males.

**Figures 17–18.**
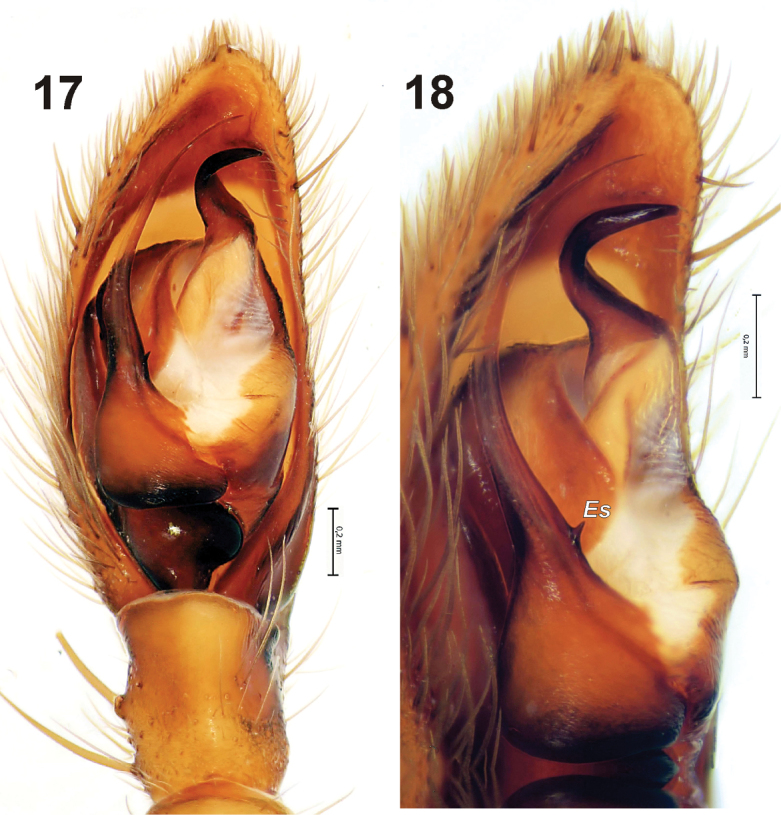
Male palp of *Gnaphosa ustyuzhanini*. **17** ventral **18** prolateral. Scale = 0.2 mm. *Es* – embolic spine.

**Figure 19.**
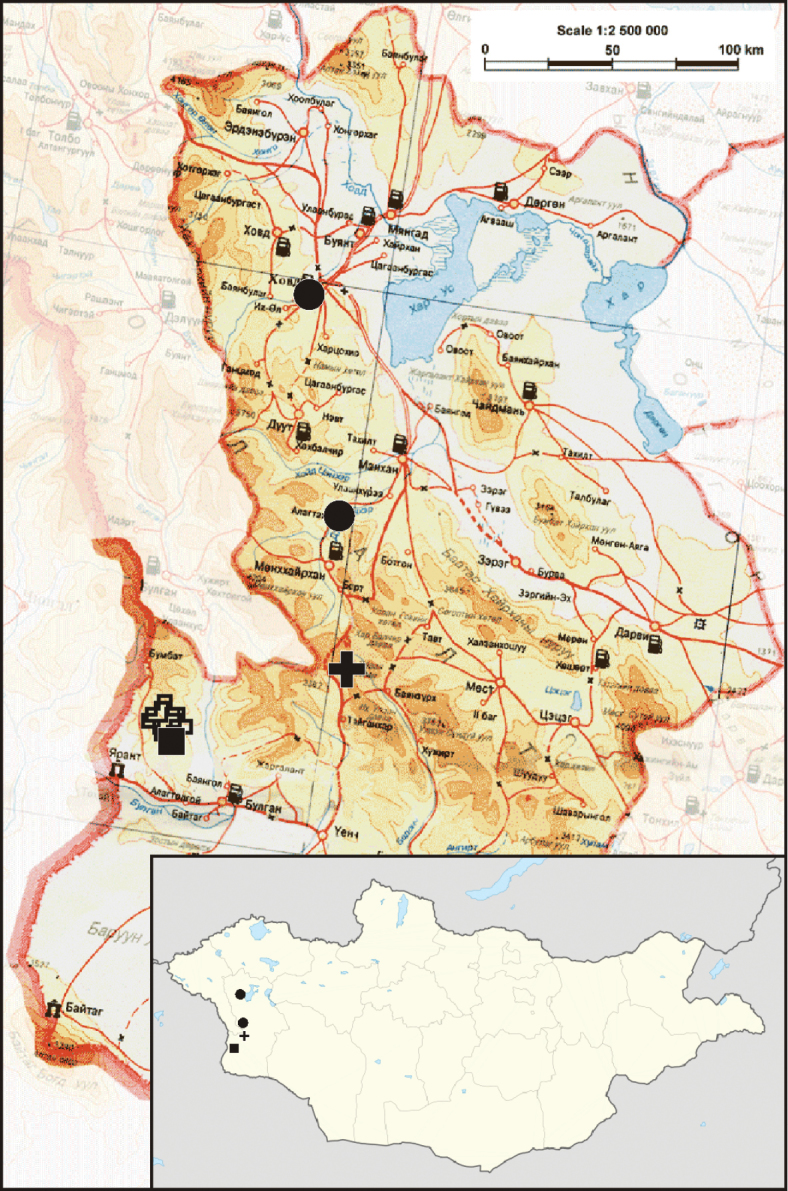
Collecting places of *Gnaphosa khovdensis* sp. n. (square), *Gnaphosa esyunini* sp. n. (circle) and *Gnaphosa ustyuzhanini* (filled cross – new locality, open crosses – earlier records).

## Discussion

Although *Gnaphosa* can be considered as a well studied genus and contains almost 150 species it was never subdivided into monophyletic groups on a global scale. Asian species have been assigned into 10 informal species groups (Ovtsharenko et al. 1992). This informal grouping was made simply to assist identification of the over 60 species known in the former USSR and Asia.

It seems that the three Mongolian species treated here, all from Khovd Aimag, form a monophyletic group, which shares the same characters such as the short tibial apophysis (not longer than tibia); raised base of embolus; base of embolus with spine in anterior part; filamentous and rounded embolus with serrated prolateral edge; and simple median apophysis with claw-like tip. Members of other Asian species groups may have any mentioned character but never all together. For example some species in the *lugubris*-group have a triangle or claw-like spine on the base of embolus, but never an anterior spine and they have a modified median apophysis with an enlarged base. Members of the *muscorum*-group have a filamentous curved embolus and short tibial apophysis, but with the base of the embolus low, never having a spine but rather a kind of spur or serrated keel. Species of the *rufula*-group have a spine or claw-like embolic tooth or nearly a spine, but have a long tibial apophysis and straight embolus, and/or modified median apophysis.

## Supplementary Material

XML Treatment for Gnaphosa khovdensis

XML Treatment for Gnaphosa esyunini

XML Treatment for Gnaphosa ustyuzhanini
